# Alexithymia and Sensory Processing Sensitivity: Areas of Overlap and Links to Sensory Processing Styles

**DOI:** 10.3389/fpsyg.2021.583786

**Published:** 2021-05-24

**Authors:** Lorna S. Jakobson, Sarah N. Rigby

**Affiliations:** Department of Psychology, University of Manitoba, Winnipeg, MB, Canada

**Keywords:** alexithymia, sensory processing sensitivity, interoception, latent profile analysis, subtype

## Abstract

Alexithymia is a dimensional trait characterized by difficulties identifying and describing feelings and an externally oriented thinking (EOT) style. Here, we explored interrelationships between alexithymia and measures assessing how individuals process and regulate their responses to environmental and body-based cues. Young adults (*N* = 201) completed self-report questionnaires assessing alexithymia, sensory processing sensitivity (SPS), interoceptive accuracy (IA), sensory processing styles, and current levels of depression, anxiety, and stress. Whereas EOT was related to low orienting sensitivity, problems with emotional appraisal (difficulties identifying feelings/difficulties describing feelings) were related to heightened sensory sensitivity. In addition, features of SPS improved the prediction of alexithymia above and beyond that accounted for by IA. We suggest that EOT is linked to problems maintaining a representation of one’s emotions in working memory and that low IA and problems with emotional appraisal are linked to atypicalities in sensory processing that may impact embodiment. A latent profile analysis revealed five classes of individuals distinguished by the relative strength of different alexithymic traits and by differences in IA and sensory processing styles. The classes identified included two lexithymic, one modal, and two alexithymic groups, showing different susceptibilities to SPS. Overall, our findings lend support to the view that alexithymia is associated with atypicalities in both bottom–up and top–down processes that impact emotion processing and regulation. They also raise the possibility that individuals with different alexithymia subtypes may differ with regard to a range of factors, including not only SPS but also early life experiences, mental health outcomes, and susceptibility to various personality disorders.

## Introduction

In a recent theoretical review, Smith et al. (2018) eloquently describe a model outlining the ways in which emotional experiences unfold and reach awareness. This process begins with one’s *affective response*—i.e., the coordinated changes that occur in one’s body and cognitive or attentional state, in response to a particular situation. Affective responses can be triggered in a bottom–up fashion or alternatively by forming or reactivating perceptual representations of current, past, or imagined situations and appraising their novelty, relevance to one’s goals or values, and controllability. Cognitive and attentional habits can bias the formation of both perceptual and appraisal-based representations, and bottom–up and top–down factors can shape higher-order representations of the affective response and its probable meaning.

Smith et al. (2018) go on to argue that the representations we form of the situation and our subsequent response to it can be “selected” for and actively maintained in working memory. Activating a *perceptual* representation of an affective response allows one to consciously experience the change in body-state that has occurred, whereas activating a *conceptual* representation of that response allows one to consciously recognize and describe the emotion that one is feeling (or the emotion of another person whose affective response has been simulated). Top–down influences determine which (if any) of the representations formed reach conscious awareness.

The ability to generate and experience emotions is clearly evolutionarily advantageous, as they motivate us to respond in ways that can help us meet our needs. However, learning to regulate emotions is also important. According to Gross (1998a,b, 2015), adults use five main strategies (alone or in combination) to manage their emotions. *Situation selection* involves actively approaching or avoiding stimuli or situations that are expected to trigger particular affective responses. The remaining strategies involve top–down control. People can physically alter the external environment in ways that make it easier for them to function well (*situation modification*). They can also use *attentional deployment*; by shifting one’s external focus (using selective attention), or by shifting attention to calming internal thoughts or mental images (engaging working memory), one can reduce the likelihood that affective responses that make one uncomfortable will reach conscious awareness. An individual might also make a *cognitive change*; for example, they might use verbal reasoning or reappraisal to change how they interpret an affective response. Finally, one can engage in *response modulation* (e.g., expressive suppression).

The models proposed by Gross (1998b) and Smith et al. (2018) can explain why our emotional experiences and our ability to self-regulate vary over time. Importantly, however, they may also help to explain individual differences in stable traits such as alexithymia. This trait, seen in approximately 10% of the general population (Mattila et al., 2010), is characterized by difficulties identifying feelings (DIF), difficulties describing feelings (DDF), an externally oriented thinking style (EOT), an impoverished fantasy life, and problems distinguishing between emotional arousal and somatic sensations (Nemiah et al., 1976). Smith et al. (2018) suggest that individuals scoring high (vs. low) on alexithymia may: (a) generate fewer or more poorly differentiated affective responses; (b) perceive/represent their affective responses in a concrete or coarse-grained manner; and/or (c) have developed a set of stable cognitive habits that make it difficult for them to exercise the top–down cognitive control that is required for them to consciously experience their affective responses and the emotions that they are feeling. Problems in any or all of these areas would make it difficult for them to identify and describe their emotions, and this, in turn, could limit their ability to understand and regulate them.

In the present study, we explored the idea that alexithymia is associated with atypicalities in a range of bottom–up and top–down processes that impact how affective responses are generated, experienced, and regulated. To do this, we collected data from a large sample of university students regarding how they process and respond to a range of body-based and environmental sensory cues and the extent to which they engage in processes *not* driven by environmental stimulation, such as visual imagery, dreaming, and some aspects of problem-solving. We then examined interrelationships between these measures using a variety of approaches. The first set of analyses were undertaken to examine general patterns seen in the sample as a whole. The second set of analyses explored the possibility that patterns of association between variables might differ across distinct subgroups of individuals.

Our study builds on past research suggesting that alexithymia is linked to atypicalities in sensory processing that could impact emotional embodiment. Much of the recent work in this area has focused on the ability to perceive internal body sensations correctly (*interoceptive accuracy* or IA). Based on their meta-analysis, Trevisan et al. (2019) concluded that there is a moderate, negative association between alexithymia and self-reported IA. Based on its links with performance on objective tests of IA, Murphy et al. (2018) argue that alexithymia may serve as an index of “multidimensional, multi-domain, interoceptive impairment” (p. 405).

Although interoceptive inputs undoubtedly contribute to embodied feelings, they cannot be the only factors that drive them, given that individuals experiencing pure autonomic failure still experience these states (Heims et al., 2004). On these grounds, we might expect alexithymia to be associated with atypicalities in the processing of a range of sensory cues. Research generally supports this idea, although the direction of effects is mixed, with some studies linking alexithymia to *exaggerated* neural, physiological, or behavioral responses to exteroceptive or body-based cues (e.g., Sivik, 1993; Nyklicek and Vingerhoets, 2000; Schafer et al., 2007; Bogdanov et al., 2013) and others linking it to *reduced* responsiveness (e.g., Pollatos et al., 2008; Goerlich-Dobre et al., 2014b; Gaigg et al., 2018). Alexithymia is also associated with atypicalities in multisensory integration, although the direction of the effects has varied (see Miles et al., 2011; Thakkar et al., 2011; Cascio et al., 2012; Germine et al., 2013; Grynberg and Pollatos, 2015; Georgiou et al., 2016). The mixed results from studies in this area could reflect the fact that researchers have not typically considered in their study designs the possibility that there may be subtypes of individuals with alexithymia who generate, experience, and regulate their emotions in different ways. Exploring this possibility is important for advancing theory and research in alexithymia.

Several studies have used the 20-item Toronto Alexithymia Scale (TAS-20; Bagby et al., 1994) in combination with other measures to look for evidence of subtypes of alexithymia. Lane et al. (2015a) distinguished anomic and agnosic forms—the former being associated with problems *naming* emotions but intact theory of mind and the latter with problems *forming conceptual representations* of emotions and impaired theory of mind. In contrast, Kajanoja et al. (2017) identified a subtype characterized by strong DIF and symptoms of depression and anxiety and another characterized by elevated DDF and EOT scores and impaired empathy.

Other subtyping studies have used the Bermond Vorst Alexithymia Scale (BVAQ; Vorst and Bermond, 2001), which samples both cognitive traits (the ability to identify, verbalize, and analyze one’s emotions) and affective traits (flattened affect and impoverished fantasy) that are associated with alexithymia. By applying factor analysis and principal component analyses to BVAQ scores, Bermond et al. (2007) identified a subtype characterized by high scores on both cognitive and affective dimensions (type I) and another characterized by high scores on the cognitive dimension but typical or unusually low scores on the affective dimension (type II). Although consensus is not universal (Bagby et al., 2009), most subtyping work using the BVAQ recognizes at least these two variants (e.g., Vorst and Bermond, 2001; Larsen et al., 2003; Berthoz and Hill, 2005; Goerlich-Dobre et al., 2014a). Some researchers describe a third subtype (type III) characterized by high scores on the affective dimension only (Bermond et al., 2006). Moormann et al. (2008) also recognized a lexithymic subtype (who score low on both dimensions), a modal subtype (who score in the average range on both dimensions), and a mixed class that does not fit into any category. They point out that because those with type III, lexithymic, and modal profiles do not have problems with emotional understanding and generally have good psychological health, it may be misleading to refer to them as alexithymia types.

Subtyping work using the BVAQ has been fruitful; however, the distinctiveness between the cognitive and affective dimensions is not always clear; indeed, there is overlap between these dimensions, particularly with regard to the “analyzing” subscale, which taps into EOT (de Vroege et al., 2018). Preece et al. (2017) also described several limitations with the BVAQ’s “emotionalizing” subscale (see also Watters et al., 2016). Another issue concerning BVAQ studies that have compared different subtypes with regard to their neural substrates (Goerlich-Dobre et al., 2015) and patterns of autonomic reactivity (Bermond et al., 2010) is that they have relied on median split procedures to create groups. The use of latent profile analysis (LPA) may be preferable, as it allows one to categorize participants in a heterogeneous sample into more homogenous subgroups based on their responses to continuous variables (Berlin et al., 2014).

In the present study, we took a unique approach to explore individual differences in alexithymia expression. Our approach was motivated by the idea (expressed by Smith et al., 2018) that two key factors contribute to the development of individual differences in people’s emotional awareness: (a) genetic/epigenetic factors and personality traits with an innate component; and (b) learning, through which cognitive habits are established. The idea that differences in these factors might result in individual differences in alexithymia led us to explore links between alexithymia and sensory processing sensitivity (SPS). SPS is a genetically predetermined trait, and its expression varies as a function of life experiences (Aron et al., 2012). SPS is characterized (in part) by a tendency to become easily overwhelmed by environmental stimuli and multitasking demands and by increased sensitivity to subtle and aesthetic features of one’s environment (Aron et al., 2012). The former feature of SPS is positively associated with DIF/DDF, and the latter is negatively related to EOT (Liss et al., 2008; Rigby et al., 2020). Unlike many people with alexithymia, individuals scoring high on SPS have a “rich” inner life, which might suggest an *enhanced* ability to keep perceptual representations of past, current, or imagined situations active in working memory. To our knowledge, no one has explored how/if two additional features of SPS—the tendency to process information at a deep/complex level and to approach novel situations cautiously—may relate to alexithymia. Doing so is of interest, as all of the features of SPS could impact how affective responses are generated, experienced, and regulated. Indeed, in adults, all three aspects of SPS are positively associated with neuroticism and negative affectivity (Lionetti et al., 2019).

Whether or not they have SPS, individuals vary in the ways in which they typically process and regulate their responses to sensory information in daily life. Given this, we were also interested in exploring how one’s *sensory processing style* might impact the expression of alexithymia. Dunn (1997) has characterized trait sensory processing styles along two dimensions: neurological threshold and behavioral regulation. The former describes the amount of sensory information required to activate the central nervous system, and the latter specifies whether an individual typically responds to sensory information actively or passively. Hyposensitivity to certain kinds of stimulation may lead to a passive failure to respond (low registration or LR) or to attempts to actively seek out stimulation (sensation seeking or Seek), whereas hypersensitivity may cause one to become easily overwhelmed (sensory sensitivity or Sen) or to actively try to avoid or reduce exposure to stimulation (sensory avoidance or SA). An individual’s sensory processing style is reflected in the *relative* strength of their Seek, SA, LR, and Sen tendencies, which can be assessed *via* a self-report measure called the Adolescent/Adult Sensory Profile (AASP; Brown and Dunn, 2002).

Relationships between alexithymic traits and AASP scores have been examined indirectly in several studies involving clinical populations. Co-occurring alexithymia has been shown to predict elevated scores on LR in adolescents with autism spectrum disorder (Milosavljevic et al., 2016). Bashapoor et al. (2015) observed that men with substance dependence showed heightened DIF and DDF, and elevated scores on LR, Seek, and SA, relative to a control group. Serafini et al. (2017) found that DIF and DDF scores were positively correlated with LR and that TAS-20 Total scores were positively associated with LR, Sen, and SA in adults diagnosed with a major mood disorder. Finally, Serafini et al. (2016) showed that AASP scores mediated the relationship between alexithymic traits and quality of life in participants with mood disorders. Although these findings suggest that alexithymia is linked to particular ways of processing and regulating one’s responses to sensory information, to our knowledge, no work has examined relationships between alexithymia and individual AASP scores (or patterns across these scores) in non-clinical samples.

In light of the discussion earlier, our first main objective was to examine interrelationships between alexithymia, SPS, and sensory processing styles in a non-clinical sample of young adults. In Part A later, we addressed three key questions: (1) “What are the relationships between alexithymia, SPS, and IA?”; (2) “Do any features of SPS improve prediction of alexithymia above and beyond that accounted for by IA?”; and (3) “Do sensory processing styles mediate the relationship between IA and specific alexithymic traits?” We expected to replicate past work demonstrating links between alexithymia and aspects of sensory processing beyond those supporting IA, but our approach allowed us to extend previous findings by considering multiple measures of sensory processing simultaneously.

Our second main objective was to test the novel prediction that subtypes of alexithymia could be distinguished based, in part, on aspects of sensory processing. Given the current interest in exploring links between alexithymia and IA (e.g., Brewer et al., 2016) and our own interest in studying aspects of one’s sensory processing style more generally, we included scores on the TAS-20 subscales, a measure of IA, and the AASP as input variables in an LPA. This analysis, described in Part B, allowed us to address two key questions: (1) “Can subtypes of individuals be identified based on their alexithymic traits, IA, and sensory processing styles?” and, if so, (2) “How do the observed subclasses differ with regard to their latent profiles?” We expected to find that individuals could be distinguished by the relative strength of different alexithymic traits and by differences in IA and sensory processing styles. We also performed planned contrasts comparing the observed classes on measures of SPS. Finally, we examined how each of the classes scored on measures of depression, anxiety, and stress, as high scores on these measures might indicate problems with emotion regulation.

## Materials and Methods

### Participants

We collected data from 209 adults recruited from the University of Manitoba’s Introduction to Psychology participant pool. One participant was excluded because she did not complete the AASP. There were no other missing data; however, seven participants were excluded because they did not achieve an acceptable score on a measure of conscientious responding (described later), suggesting that they were exhibiting poor effort. This left a final sample of 201 (112 women and 89 men, *M*_*age*_ = 19.7 years, *SD* = 3.9, range 17–52). An *a priori* power analysis suggested this would be more than sufficient to detect a medium effect (*f*^2^ = 0.15) in the planned hierarchical regression described later with a power of 0.80. We over-recruited to achieve a large enough sample for the LPA. To our knowledge, there are no firm guidelines regarding power and sample size requirements in LPA (Nylund-Gibson and Choi, 2018). However, results from a latent class analysis simulation study with *N* = 200 (Nylund et al., 2007) and findings from Tein et al. (2013) suggest that our final sample size, while on the low side, was likely adequate. Participants received credit toward a course requirement for taking part.

### Procedures

Participants were tested in a computer lab in groups of approximately 30. They provided informed consent, indicated their age and their biological sex, and then completed several self-report measures. Items comprising the TAS-20 (Bagby et al., 1994), the Highly Sensitive Person Scale (HSPS; Aron and Aron, 1997), the Orienting Sensitivity (OS) subscale of the Adult Temperament Questionnaire—Short (Evans and Rothbart, 2007), the Interoceptive Accuracy Scale (IAS; Murphy et al., 2019), the Depression, Anxiety and Stress Scale (DASS-21; Lovibond and Lovibond, 1995), and the Conscientious Responders Scale (CRS; Marjanovic et al., 2014) were presented and responded to *via* an online Qualtrics survey. The AASP (Brown and Dunn, 2002) was administered in paper format. Half of the participant groups completed the Qualtrics survey first; the other half began with the AASP. The testing protocol was approved by the Psychology/Sociology Human Research Ethics Board at the University of Manitoba.

### Materials

#### Toronto Alexithymia Scale (TAS-20)

The 20 items comprising the TAS-20 tap into three of the core features of alexithymia: DIF (seven items), DDF (five items), and EOT (eight items). Participants respond to each item on a five-point Likert scale ranging from 1 = *Strongly disagree* to 5 = *Strongly agree*; thus, total scores can range from 20 to 100. According to the convention, scores ≥61 signify alexithymia, scores ≤51 signify lexithymia, and scores falling between these cut points are classified as borderline (Parker et al., 2003). However, in line with recommendations by Bagby et al. (2020), we treated alexithymia as a dimensional, rather than a categorical, construct in our analyses.

#### Sensory Processing Sensitivity

We used two complementary measures to assess SPS: the HSPS and the OS scale. The total (mean) score on the 27-item HSPS provides a general measure of SPS. These scores are approximately normally distributed and can be used to classify individuals into three sensitivity groups: low (≤30th percentile), medium (between 30th and 70th percentile), and high (≥70th percentile) (Lionetti et al., 2018). By convention, these groups are referred to as Dandelions, Tulips, and Orchids, respectively.

Three HSPS subscale scores can be derived by averaging responses across relevant items (Lionetti et al., 2018). The ease-of-excitation (EOE) score reflects an individual’s tendency to become overwhelmed by sensory cues, and the low sensory threshold (LST) score taps into the extent to which someone experiences unpleasant sensory arousal in response to subtle environmental stimulation. Thus, together, these scales focus on how affected one is by different types of sensory stimuli and how one characteristically responds to them (e.g., whether one finds multitasking challenging or avoids watching violent television shows). Finally, the aesthetic sensitivity (AES) score reflects the extent to which one appreciates aesthetic features of the environment (e.g., music and the arts).

Aron et al. (2012) recommend supplementing the HSPS with the OS scale. The 15 items comprising this measure are responded to using a seven-point Likert scale, ranging from 1 = *Extremely untrue of you* to 7 = *Extremely true of you*. The OS scale yields a total score and three subscale scores: Neutral Perceptual Sensitivity (NPS; five items), Affective Perceptual Sensitivity (APS; five items), and Associative Sensitivity (AS; five items). The NPS subscale taps into one’s awareness of low-intensity environmental cues transmitted through the visual, auditory, tactile, and olfactory-gustatory modalities (e.g., the extent to which you notice other people’s eye color). The APS subscale taps into awareness of how low-intensity environmental cues (e.g., a room’s color/lighting), including those conveyed through music or the visual arts, affects one’s mood; as such, it shares some overlap with the AES scale from the HSPS. Finally, the AS subscale taps into the extent to which one engages in processes that are *not* driven by stimuli in the immediate environment, such as some aspects of problem-solving, vivid imagery, and dreaming; thus, it captures aspects of depth of processing and the richness of one’s inner life that are not assessed by the HSPS but that characterize those with SPS.

#### Interoceptive Accuracy Scale

The IAS is a unidimensional self-report measure that correlates with objectively measured IA; as such, it is purported to provide a general index of IA functioning (Murphy et al., 2019). It comprises 21 items that tap into one’s perception of a wide range of bodily sensations (e.g., “I can always accurately perceive when I am hungry”). Participants respond to each item using a five-point Likert scale ranging from 1 = *Strongly agree* to 5 = *Disagree strongly*. Scores can range from 21 to 105, with higher scores representing better IA. The IAS exhibits good internal consistency, test–retest reliability, and construct validity (Murphy et al., 2019).

#### Depression, Anxiety and Stress Scale (DASS-21)

The DASS-21 is a self-report measure designed to assess levels of depression, anxiety, and stress experienced over the past week. Each of the three subscales includes seven items that are responded to on a scale from 0 = *Did not apply to me at all* to 3 = *Applied to me very much or most of the time.* Scores on relevant items are added, and the sum is then multiplied by two to obtain each subscale score. The manual (Lovibond and Lovibond, 1995) provides recommended cutoff scores that can be used to determine the severity of each symptom.

#### Attention Checks: Conscientious Responders Scale

The CRS (Marjanovic et al., 2014) is a measure designed to assist researchers in detecting poor effort in participants’ responding during surveys. The five items comprising the CRS were randomly dispersed throughout the other items included in the Qualtrics survey. Each CRS item instructs participants to respond in a particular way—for example: *To respond to this question, please choose option number five, “slightly agree.”* There are five response options for each item, and responses are scored as correct or incorrect. In accordance with the recommendations of the authors of the scale, scores ≥3 are taken as evidence of sufficiently conscientious responding.

#### Adolescent/Adult Sensory Profile (AASP)

The AASP (Brown and Dunn, 2002) consists of 60 items that measure trait sensory processing styles in daily life. There are 15 items assessing each of the four quadrants defined in the model by Dunn (1997): LR, Seek, Sen, and SA. LR items tap into the extent to which an individual fails to notice or is slow to respond to environmental stimuli. Seek items focus on one’s proclivity to seek out and enjoy environmental stimulation. SA items inquire about attempts to avoid or reduce exposure to environmental stimuli. Finally, Sen items assess the degree to which one notices and is distracted or made uncomfortable by environmental stimuli. Participants respond to each item using a five-point Likert scale, ranging from 1 = *Almost never* to 5 = *Almost always*. Individual quadrant scores can range from 5 to 75. The items comprising the AASP tap into responses to visual, auditory, tactile, and taste/smell cues, as well as vestibular/proprioceptive cues related to movement processing, and Brown and Dunn report that quadrant scores generalize across these different sensory modalities.

Brown et al. (2001) conducted a large standardization study that included 950 adolescents/adults. They reported that the AASP has good convergent and discriminant validity. They also reported that the internal consistency of quadrant scores ranged from an alpha of 0.64 to 0.78. Based on the results of their standardization study, Brown et al. (2001) provided cut-scores clinicians can use to identify unusually high or low quadrant scores. Later, we refer to quadrant scores for our participants that fell above the 84th and below the 16th percentile of a normative sample in Brown et al. (2001) as *peaks* and *valleys*, respectively.

## Results and Discussion

Descriptive statistics for the study variables are presented in Table 1. In Parts A and B, we present results and preliminary discussion of the two sets of analyses that were carried out. Each section is organized around the specific research questions articulated at the end of *Introduction*, which are reprinted later. Analyses were completed using SPSS 25 (IBM Corp., Armonk, NY, United States) and MPlus version 6.0 (Muthén and Muthén, 1998-2017). Unless otherwise indicated, an alpha level of 0.05 was adopted for tests of significance.

**TABLE 1:** Descriptive statistics for study variables in the full sample (*N* = 201).

		*M* (*SD*)	Minimum	Maximum
TAS-20	Total	53.9 (10.6)	29	82
	DIF	18.5 (5.8)	7	34
	DDF	15.5 (4.6)	5	25
	EOT	19.9 (4.2)	8	31
HSPS	Total	4.0 (0.8)	1	7
	EOE	4.4 (1.0)	1	7
	AES	4.4 (0.9)	2	6
	LST	3.2 (1.3)	1	7
OS	Total	72.0 (10.6)	44	102
	NPS	23.6 (4.2)	12	35
	APS	23.8 (5.3)	8	35
	AS	24.7 (4.9)	11	35
IAS	Total	83.2 (10.0)	58	105
AASP	Seek	47.4 (7.3)	29	65
	LR	34.0 (6.8)	19	57
	Sen	38.8 (8.4)	17	66
	SA	40.0 (8.0)	22	61
DASS-21	Depression	12.0 (8.8)	0	42
	Anxiety	12.5 (7.7)	0	36
	Stress	14.1 (7.4)	0	34

*TAS-20, Toronto Alexithymia Scale; DIF, Difficulty Identifying Feelings; DDF, Difficulty Describing Feelings; EOT, Externally Oriented Thinking; HSPS, Highly Sensitive Person Scale; EOE, Ease of Excitation; AES, Aesthetic Sensitivity; LST, Low Sensory Threshold; OS, Orienting Sensitivity; NPS, Neutral Perceptual Sensitivity; APS, Affective Perceptual Sensitivity; AS, Associative Sensitivity; IAS, Interoceptive Accuracy Scale; AASP, Adult/Adolescent Sensory Profile; Seek, Sensation Seeking; LR, Low Registration; Sen, Sensory Sensitivity; SA, Sensory Avoidance. DASS-21, Depression, Anxiety and Stress Scale.*

### Part A: Relationships Between Variables in the Full Sample

**Question 1. What are the relationships between alexithymia, SPS, and IA?** As a first step, we performed correlational analyses to explore the relationships between measures of alexithymia, SPS, and IA, after controlling for participant age. Benjamini–Hochberg adjusted *p* values were computed to control for multiple comparisons and evaluated for significance using a false discovery rate of 0.05.

**FIGURE 1:**
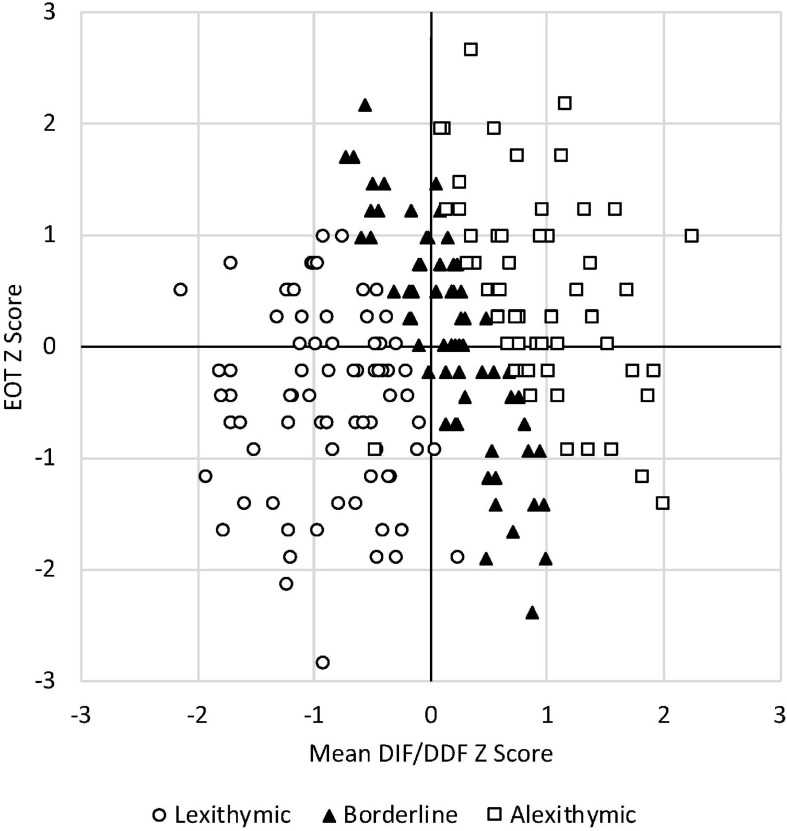
Individual points show the relative strength of EOT and DIF/DDF across the full sample (*N* = 201). Values are expressed as *Z* scores. High scores on the vertical axis indicate stronger EOT, and high scores on the horizontal axis indicate greater problems identifying and describing one’s feelings. Assignment to lexithymic, borderline, and alexithymic subgroups was based on established cut scores (Parker et al., 2003).

We report here the analyses conducted on the full sample, as exploratory analyses confirmed that the relationships described later held in both men and women. As can be seen in Table 2, scores on the DIF and DDF subscales were strongly related to one another but not to scores on the EOT subscale. This supports the view that problems with emotional appraisal (high DIF/DDF) and EOT are fundamentally different (see also Preece et al., 2017). It also suggests that in a general sample, there is likely considerable variation in where individuals score in these two areas. This was seen in our data. In Figure 1, we have plotted individual participant’s *Z* scores on the EOT subscale against the mean of their DIF and DDF *Z* scores (which was strongly correlated with both subscale scores, *r* = 0.89). The figure shows that obtaining almost any combination of the EOT and DIF/DDF *Z* scores is possible, but an important takeaway is that problems with emotional appraisal are the *sine qua non* of alexithymia. Thus, although individuals scoring in what is traditionally considered the alexithymic range on the TAS-20 (i.e., ≥61; represented by squares in the figure) had EOT scores that varied widely (from −1.45 to +2.68 *SD* from the sample mean), their DIF/DDF scores were consistently above average.

**TABLE 2:** Intercorrelations between measures of alexithymia, sensory processing sensitivity, and interoceptive accuracy.

		TAS-20				HSPS				OS			
		Total	DIF	DDF	EOT	Total	EOE	AES	LST	Total	NPS	APS	AS
TAS-20	Total	−											
	DIF	0.84*	−										
	DDF	0.80*	0.58*	−									
	EOT	0.50*	0.09	0.12	−								
HSPS	Total	0.35*	0.47*	0.34*	–0.14	−							
	EOE	0.41*	0.49*	0.37*	–0.04	0.89*	−						
	AES	0.05	0.23*	0.15*	−0.37*	0.65*	0.36*	−					
	LST	0.23*	0.29*	0.20*	–0.04	0.80*	0.57*	0.37*	−				
OS	Total	–0.04	0.14	0.08	−0.37*	0.37*	0.16*	0.61*	0.25*	−			
	NPS	–0.14	–0.09	–0.04	−0.17*	0.00	–0.08	0.16*	0.00	0.60*	−		
	APS	–0.06	0.12	0.01	−0.32*	0.44*	0.25*	0.60*	0.31*	0.82*	0.25*	−	
	AS	0.11	0.25*	0.19*	−0.30*	0.33*	0.15	0.54*	0.20*	0.77*	0.17*	0.47*	–
IAS	Total	−0.27*	−0.20*	−0.17*	−0.21*	–0.06	–0.11	0.11	–0.07	0.22*	0.15	0.29*	0.05

TAS-20, Toronto Alexithymia Scale; DIF, Difficulty Identifying Feelings; DDF, Difficulty Describing Feelings; EOT, Externally Oriented Thinking; IAS, Interoceptive Accuracy Scale; HSPS, Highly Sensitive Person Scale; EOE, Ease of Excitation; AES, Aesthetic Sensitivity; LST, Low Sensory Threshold; OS, Orienting Sensitivity; NPS, Neutral Perceptual Sensitivity; APS, Affective Perceptual Sensitivity; AS, Associative Sensitivity. Values shown are partial correlations, controlling for age. *Benjamini–Hochberg Adjusted *p*-value significant (0.0001 ≤ *p* ≤ 0.03), using False Discovery Rate = 0.05.

TAS-20 total scores showed a moderately strong, positive relationship with HSPS total scores; this reflected, in part, the fact that (unlike EOT scores) DIF and DDF scores were positively related to EOE and, to a lesser extent, LST scores. These results replicate earlier findings (Liss et al., 2008; Rigby et al., 2020) and suggest that many individuals who have difficulties with emotional appraisal report being easily overwhelmed or made uncomfortable by sensory stimulation. This heightened sensitivity could interfere with emotional appraisal and regulation in a variety of ways. For example, it might disrupt embodiment (in a bottom–up fashion) or make it difficult for someone to link the response that they *consciously* experience with the stimulus that triggered that response or with their current goal.

Unlike DIF and DDF, EOT scores were negatively related to AES and OS subscale scores. The moderate negative relationship between EOT and scores on the AES and APS subscales suggests that those who score high on EOT are less aware of how subtle (aesthetic) features of their surroundings affect their mood. This could reflect an underlying problem selecting and maintaining a (weak) representation in working memory. In addition to making it difficult to *attend* to one’s feelings, problems in this area could limit one’s ability to experience vivid mental images (and, potentially, to use these to distract oneself from unpleasant situations). The latter point is noteworthy given that EOT was also negatively correlated with scores on the AS subscale, which taps into imagery and dreaming. Indeed, Bagby et al. (2020) suggest that the EOT subscale indirectly assesses fantasy.

Previous work suggests that alterations in interoceptive abilities are characteristic of people with alexithymia (e.g., Murphy et al., 2018). Consistent with other reports (e.g., Murphy et al., 2019), we found that people who scored lower on IA had higher TAS-20 scores (see Table 2). Additionally, we found that individuals with low IA reported reduced sensitivity to low-intensity cues that define the aesthetic qualities of the environment (APS). However, because IA scores were *unrelated* to scores on the AS subscale (which taps into the richness of one’s inner life), we suggest that low IA might arise primarily from atypical sensory processing/integration that impacts embodiment, rather than from general difficulties selecting and maintaining representations in working memory.

The foregoing illustrates why, when exploring relationships between alexithymia and SPS, it is important to examine the subscales individually rather than relying exclusively on total scores. It also confirms the importance of supplementing the HSPS with the OS to capture the full range of traits associated with SPS (as recommended by Aron et al., 2012). Had we not done this, we would not have noted the strong, negative relationship between EOT and scores on the OS scale, generated testable hypotheses regarding how specific aspects of alexithymia relate to working memory and visual imagery, or noted the link between low IA and reduced orienting sensitivity.

**Question 2. Do any features of SPS improve the prediction of alexithymia above and beyond that accounted for by IA?** This question is important, given the strong focus in the current literature on the role of interoceptive impairment in alexithymia. To address this question, we ran a hierarchical multiple regression using the forced entry method. We entered IA at step 1 and subscale scores for the HSPS and OS scales as predictors at step 2. Multicollinearity was not an issue (VIF ≤ 1.97 for all predictors). As shown in Table 3, both models were significant, as was the change in *R*^2^ at Step 2 (*f*^2^ = 0.126 [95% CI: 0.034, 0.236]). IA continued to predict TAS-20 total scores following the introduction of the SPS measures, but EOE and AS also accounted for unique variance. Scoring low in IA and/or high in EOE or AS was associated with reporting stronger signs of alexithymia overall. [These same predictors were also significant in exploratory regressions run on females only. The same *pattern* was also seen in males, but in this case, IAS, EOE, and NPS scores emerged as the significant predictors in Model 2. In both females and males, however, the general conclusion (i.e., that characteristics associated with SPS improve prediction of TAS-20 scores above-and-beyond IA) was still supported.]

**TABLE 3:** Hierarchical model of predictors of TAS-20 total scores.

			*b*	*SE b*	β	*p*
Model 1	*F*(1,200) = 15.47***	(Constant)	77.653	6.077		
	*R*^2^ = 0.072***	IAS Total	−**0.285**	**0.073**	−**0.269**	**<0.001**
Model 2	*F*(7,200) = 9.08***	(Constant)	55.061	7.768		
	△*R*^2^ = 0.176***	IAS Total	−**0.183**	**0.071**	−**0.173**	**0.011**
		EOE	**0.378**	**0.070**	**0.424**	**<0.001**
		LST	−0.006	0.109	−0.004	0.954
		AES	−0.161	0.147	−0.096	0.277
		NPS	−0.169	0.167	−0.066	0.313
		APS	−0.243	0.171	−0.122	0.156
		AS	**0.368**	**0.167**	**0.169**	**0.029**

IAS, Interoceptive Accuracy Scale. Subscales from the Highly Sensitive Person Scale: EOE, Ease of Excitation; LST, Low Sensory Threshold; AES, Aesthetic Sensitivity. Subscales from the Orienting Sensitivity Scale of the Adult Temperament Questionnaire (Short): NPS, Neutral Perceptual Sensitivity; APS, Affective Perceptual Sensitivity; AS, Associative Sensitivity. Bolded values are significant at the *p* < 0.05 level or below. ***p < 0.001.

The findings described earlier suggest that low IA may be just one facet of atypical sensory processing that can characterize people with alexithymia and that some individuals with alexithymia may have co-occurring SPS. Indeed, in a recent study involving 106 undergraduate students, we found that close to 50% of those who scored in the upper third of the distribution of TAS-20 scores could be classified as Orchids based on their HSPS scores (Rigby et al., 2020).

**Question 3. Do sensory processing styles mediate the relationship between IA and specific alexithymic traits?** To determine if one’s characteristic *sensory processing style* mediates the relationship between IA and specific alexithymic traits, we tested a model in which the four AASP quadrant scores were entered as correlated mediators of the links between IA and TAS-20 subscale scores. Mediation fit statistics indicate a good fit if the comparative fit index (CFI) is ≥0.95, the Standardized Root Mean Square Residual (SRMR) is ≤0.08, and the root mean square error of approximation is ≤0.05 (Hu and Bentler, 1999; Gunzler et al., 2013). Based on these indices, the model exhibited good fit: χ^2^(3) = 5.63, *p* = 0.13; CFI = 0.99; SRMR = 0.024; root mean square error of approximation = 0.066.

**FIGURE 2:**
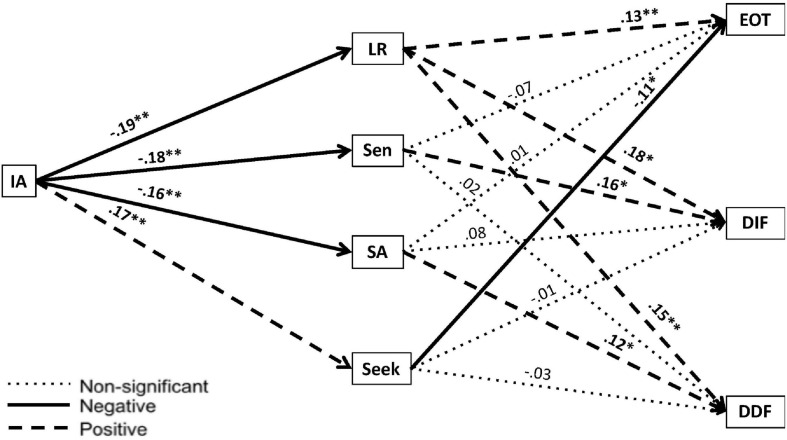
Mediation model evaluating the indirect effects of sensory profile quadrant scores as correlated mediators on the relationship between interoceptive accuracy and the subscales of the TAS-20. IA, Interoceptive Accuracy; Adolescent/Adult Sensory Profile; LR, Low Registration; Sen, Sensory Sensitivity; SA, Sensation Avoidance; TAS-20, Toronto Alexithymia Scale – 20. ^∗^*p* < 0.05; ^∗∗^*p* < 0.01.

As shown in Figure 2, we observed significant indirect effects of IA on EOT through LR (*B* = −0.03, 95% CI [−0.05, −0.01]) and Seek (*B* = −0.02, 95% CI [−0.04, −0.004]). Thus, low IA was linked to high EOT because both were associated with failing to notice (high LR) and failing to seek out (low Seek) sensory stimulation. These associations make sense, given that these behavioral tendencies could be driven by bottom–up problems with sensory processing/integration (associated with low IA) and/or by top–down problems with working memory that limit one’s conscious experience of emotions (associated with EOT).

We also observed significant indirect effects of IA on DIF through LR (*B* = −0.04, 95% CI [−0.07, −0.01]) and Sen (*B* = −0.03, 95% CI [−0.07, −0.004]) and significant indirect effects of IA on DDF through LR (*B* = −0.03, 95% CI [−0.06, −0.01]) and SA (*B* = −0.02, 95% CI [−0.05, −0.002]). Thus, low IA was linked to problems with emotional appraisal because both were associated with scoring high on LR and with using regulatory strategies to deal with sensory sensitivity that could be dysfunctional if carried to an extreme (high SA and Sen). Given that LR is associated with hyposensitivity and SA and Sen with hypersensitivity, it may seem counterintuitive that some individuals scoring in the alexithymic range showed elevated scores in all three of these AASP quadrants. We return to this point in Part B and offer some possible explanations for this, gleaned from a careful examination of individual participants’ AASP profiles and other observations. For now, we will simply highlight that associations between alexithymia and high LR, SA, and Sen scores have also been noted in various clinical groups (Bashapoor et al., 2015; Milosavljevic et al., 2016; Serafini et al., 2016; Engel-Yeger et al., 2018).

### Part B: Subtyping

The results of our mediation analysis support the idea that alexithymic traits show unique relationships with certain aspects of sensory processing and self-regulation. In Part B, we sought to determine whether patterns in these relationships vary across distinct subgroups of individuals. Once again, we have organized the presentation of our results and discussion around several key questions.

**Question 1. Can subtypes of individuals be identified based on their alexithymic traits, IA, and sensory processing styles?** We utilized LPA to address this question, including subscale scores on the TAS-20, the IAS, and the AASP quadrant scores as input variables. Selecting the optimal number of classes to fit the data is a complicated task, and when doing so, the researcher should consider the particular research question, theory based on previous research, the meaning of the model, and observed fit statistics (see Berlin et al., 2014). As recommended by Yang (2006), we first carefully compared the latent profiles of classes identified in each model tested with regard to their theoretical meaningfulness and distinctiveness. Next, we compared the models on a variety of fit statistics. One of these was entropy, which provides a standardized measure of classification accuracy (see Berlin et al., 2014); higher entropy values indicate a better fitting model (Wang et al., 2017). The remaining fit statistics we examined were the sample size adjusted Bayesian Information Criterion (ABIC; Sclove, 1987) and the approximate *p*-value for the Bootstrapped Likelihood Ratio Test (BLRT; McLachlan and Peel, 2000). Smaller ABIC values indicate a better fit. The BLRT test is used to compare the improvement in fit between models; here, statistically significant *p*-values indicate a better fit for the current (*k*) model than the preceding (*k−*1) model.

We began by testing a two-class model and then increased the number of classes by one until the best fitting model was identified. As can be seen in Table 4, overall, the models tested had good classification quality. Although entropy remained high and relatively stable across the 3- to 6-class models, ABIC values and the BLRT results indicated that each successive model provided a better fit than the one before. One could argue that the six-class model provided the best fit to the data on statistical grounds; however, we retained the five-class model for two key reasons. First, as we will show later, the latent class profiles for the five-class model were more distinctive and theoretically meaningful than those of the four-class model (indeed, two of the classes in the four-class model differed only with regard to LR, Sen, and SA scores). Second, the six-class model included one class that was quite small (6% of the total sample), suggesting possible overfitting (Wang and Wang, 2012; Wickrama et al., 2016).

**TABLE 4:** Fit statistics for 2- to 6-class latent profile models (*N* = 201).

Model	ABIC	Change in ABIC	Entropy	BLRT *p*-value
2-Class	10498.05	0.00	0.72	<0.001
3-Class	10447.52	−50.53	0.80	<0.001
4-Class	10424.24	−23.28	0.81	<0.001
**5-Class**	**10407.59**	−**16.65**	**0.79**	**<0.001**
6-Class	10393.03	−14.56	0.79	<0.001

*ABIC, sample size adjusted Bayesian Information Criterion; BLRT, Bootstrapped Likelihood Ratio Test. Five-class model (shown in bold) was selected as the best-fitting model.*

Descriptive information about the latent classes in the five-class model is shown in Table 5. The sex distributions varied somewhat across classes, χ^2^(4) = 11.50, *p* = 0.022, but only class 4 included a significantly higher proportion of women than men (72 vs. 28%; one-sample binomial test, *p* = 0.01). The table shows the proportion of participants in each class who met traditional criteria for being classified as lexithymic, borderline, or alexithymic based on TAS-20 total scores (Parker et al., 2008) and the proportion who would be classified as Dandelions, Tulips, or Orchids based on HSPS Total scores (as per Lionetti et al., 2018). For descriptive purposes, we present the mean total and subscale scores for the HSPS and the OS scale for each class, expressed as *Z-*scores based on the distribution of each variable in the full sample (*N* = 201). Mean *Z*-scores for the DASS-21 subscales are also shown, along with the percentage of individuals in each class who reported low, mild, moderate, severe, and very severe signs of depression, anxiety, and stress (based on cut-scores recommended by the scale developers). [Note that one expects 50% of people to score within 0.66 SD of the mean; as such, following convention, we refer to *Z* scores falling within this range as “average” scores.]

**TABLE 5:** Characteristics related to alexithymia, SPS, and mental health in the five latent classes.

		Class 1	Class 2	Class 3	Class 4	Class 5

		Lexithymic Orchids	Lexithymic Dandelions	Modal	Alexithymic Tulips	Alexithymic Orchids
Sample size		21	25	89	39	27
(% of total sample)		(10.4%)	(12.4%)	(44.3%)	(19.4%)	(13.4%)
Sex distribution (% female)		52.4	32.0	52.8	71.8	66.7
TAS type^a^	% Lexithymic	90.5	100.0	34.8	2.6	7.4
	% Borderline	9.5	0.0	40.4	43.6	40.7
	% Alexithymic	0.0	0.0	24.7	53.8	51.9
HSPS type^a^	% Dandelion	14.3	84.0	37.1	10.3	0.0
	% Tulip	28.6	8.0	48.3	59.0	22.2
	% Orchid	57.1	8.0	14.6	30.8	77.8
HSPS	Total *Z*	0.50	−1.19	−0.24	0.33	1.04
	EOE *Z*	0.29	−1.21	−0.11	0.27	0.86
	AES *Z*	0.26	−0.48	−0.16	−0.07	0.86
	LST *Z*	0.77	−0.89	−0.37	0.51	0.71
OS	Total *Z*	0.43	0.13	−0.20	−0.25	0.56
	NPS *Z*	0.31	0.45	−0.10	−0.23	0.00
	APS *Z*	0.49	0.05	−0.22	−0.21	0.60
	AS *Z*	0.13	−0.15	−0.11	−0.10	0.55
DASS-21	*Z*	−0.23	−0.99	0.01	0.34	0.58
Depression	% low	57	88	42	21	22
	% mild	19	4	18	26	19
	% moderate	14	4	24	38	30
	% severe	10	4	15	8	15
	% very severe	0	0	2	8	15
DASS-21	*Z*	−0.25	−0.80	−0.06	0.29	0.72
Anxiety	% low	24	64	34	5	11
	% mild	5	8	8	8	7
	% moderate	52	16	24	41	26
	% severe	19	8	16	28	11
	% very severe	0	4	19	18	44
DASS-21	*Z*	0.01	−1.10	−0.02	0.20	0.80
Stress	% low	48	92	58	44	30
	% mild	38	8	18	33	11
	% moderate	14	0	19	18	33
	% severe	0	0	4	5	19
	% very severe	0	0	0	0	7

*HSPS, Highly Sensitive Person Scale; EOE, Ease of Excitation; AES, Aesthetic Sensitivity; LST, Low Sensory Threshold; OS, Orienting Sensitivity Scale; NPS, Neutral Perceptual Sensitivity; APS, Affective Perceptual Sensitivity; AS, Associative Sensitivity. DASS-21, Depression, Anxiety and Stress Scale. *Z-*scores are means for a given class and are based on the distribution of scores in the full sample (*N* = 201). The “average” range for mean *Z-*scores was considered to be −0.66 to 0.66. ^*a*^TAS and HSPS types were identified following standard practices (Parker et al., 2003; Lionetti et al., 2018).*

Classes 1 and 2 were overwhelmingly lexithymic. They differed in that class 1 scored significantly above and class 2 significantly below the mean for the HSPS Total score. The majority of those in class 1 were Orchids, and the majority of those in Class 2 were Dandelions. Most individuals in class 3 had TAS-20 scores that put them in, or close to, the borderline range. Their mean HSPS and OS total scores were close to the sample mean, and they were most frequently classified as Tulips. Classes 4 and 5 ncluded the largest proportion of alexithymic individuals, but whereas the majority of those in class 5 were Orchids. To recap, the LPA revealed five theoretically meaningful subtypes of individuals: two lexithymic and two alexithymic classes with differing levels of SPS and a group that scored in the mid-range with regard to both traits. To capture the distinguishing features of the different classes, hereafter, we refer to class 1 as *Lexithymic Orchids*, class 2 as *Lexithymic Dandelions*, class 3 as *Modal*, class 4 as *Alexithymic Tulips*, and class 5 as *Alexithymic Orchids*. Between-class differences in self-reported depression, anxiety, and stress are discussed later.

**FIGURE 3:**
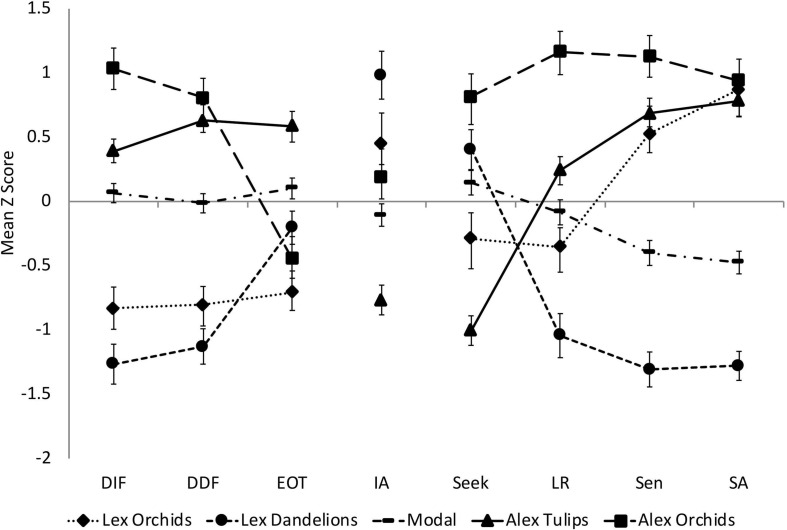
Mean *Z*-scores (SEs indicated) for each class on the input variables, which included the Toronto Alexithymia Scale (TAS-20) subscales: Difficulty Identifying Feelings (DIF), Difficulty Describing Feelings (DDF), Externally Oriented Thinking (EOT); the Interoceptive Accuracy (IA) scores; and the Adolescent/Adult Sensory Profile quadrant scores: Sensation Seeking (Seek), Low Registration (LR), Sensory Sensitivity (Sen), and Sensory Avoidance (SA).

**Question 2. How do the observed subtypes differ with regard to their latent profiles?** To compare the latent profiles of the five classes, input variables were converted to *Z*-scores (using data from the full sample) to place them on a common scale and then plotted (see Figure 3). We next ran a series of *post hoc* tests on the *Z* scores to explore how the classes differed with respect to these variables. Because the five classes had unequal sample sizes, we used Kruskal–Wallis tests to determine if they differed from one another in their scores on each variable (see Table 6 for *H* statistics). In addition, we used repeated-measures analyses of variance to determine if *Z* scores for individual subscales of the TAS-20 and the AASP differed *within* a given class (see Table 6 for *F* statistics). In both sets of analyses, Bonferroni corrections were made for all *post hoc* pairwise comparisons (adjusted *p* values for specific contrasts are provided in the main text). Later, we describe the latent profile of each class and highlight some important similarities and differences between particular classes.

**TABLE 6:** Within- and between-class comparisons for LPA input variables in the five class model.

Measure	Value displayed	Class 1 Lexithymic Orchids	Class 2 Lexithymic Dandelions	Class 3 Modal	Class 4 Alexithymic Tulips	Class 5 Alexithymic Orchids	Kruskal– Wallis *H ^a^*
TAS-20	*M* DIF (*SE*)	−0.83(0.14)	−1.27(0.14)	0.07(0.08)	0.39(0.12)	1.03(0.15)	86.9***
	*M* DDF (*SE*)	−0.80(0.16)	−1.13(0.13)	−0.01(0.09)	0.63(0.12)	0.81(0.17)	77.3***
	*M* EOT (*SE*)	−0.70(0.24)	−0.20(0.19)	0.10(0.10)	0.59(0.12)	−0.44(22)	30.8***
	ANOVA^*a*^ *F*($\eta_{\text{p}}^{2}$)	0.16(0.008)	21.4(0.471)***	0.48(0.005)	1.07(0.027)	21.8(0.456)***	
IAS	*M* IAS (*SE*)	0.45(0.20)	0.98(0.16)	−0.11(0.10)	−0.77(0.11)	0.19(0.18)	53.6***
AASP	*M* Seek (*SE*)	−0.29(0.15)	0.40(0.17)	0.15(0.10)	−1.00(0.10)	0.82(0.16)	66.2***
	*M* LR (*SE*)	−0.35(0.21)	−1.04(0.14)	−0.08(0.09)	0.24(0.12)	1.16(0.16)	66.7***
	*M* Sen (*SE*)	0.53(0.12)	−1.31(0.11)	−0.40(0.06)	0.69(0.12)	1.13(0.17)	118.4***
	*M* SA (*SE*)	0.87(0.13)	−1.28(0.11)	−0.47(0.06)	0.78(0.10)	0.94(0.15)	134.5***
	ANOVA^*a*^ *F*($\eta_{\text{p}}^{2}$)	16.6(0.454)***	42.0(0.636)***	14.5(0.142)***	70.4(0.650)***	1.07(0.040)	

Values shown for LPA input variables are mean *Z*-scores (*SE* indicated in brackets). TAS-20, Toronto Alexithymia Scale; DIF, Difficulty Identifying Feelings; DDF, Difficulty Describing Feelings; EOT, Externally Oriented Thinking subscales. IAS, Interoceptive Accuracy Scale. AASP, Adolescent/Adult Sensory Profile; Seek, Sensation Seeking; LR, Low Registration; Sen, Sensory Sensitivity; SA, Sensory Avoidance subscales. ^*a*^Between-class comparisons were performed using Kruskal–Wallis *H* tests; within-class comparisons were performed using repeated-measures analysis of variance (ANOVA) tests. ***p < 0.001.

#### Modal Group

The Modal group exhibited average scores on all of the input variables, but their Sen and SA scores were significantly lower than their LR and Seek scores (*p* ≤ 0.023). Thus, the largest class of individuals (*n* = 89) in this university sample reported a slightly below-average tendency to be overwhelmed by and to avoid sensory stimulation. Their DASS-21 scores were close to the sample mean (Table 5), with the majority of individuals reporting low-to-mild symptoms of depression, anxiety, and stress. This group may be similar to the “Modal” type described by Moormann et al. (2008), who scored in the average range on both the cognitive and affective composites of the BVAQ and had generally good mental health.

#### Lexithymic Dandelions

The Lexithymic Dandelions scored low on DIF/DDF, but their EOT scores were near the sample mean, and their IA scores were above average. Their AASP scores were uneven and unique in several respects. First, this was the only class in whom Seek was the highest of the quadrant scores. Second, this was the only class in whom we saw extremely low scores (valleys) in the SA and/or Sen quadrants; this pattern was evident in one-third of cases. Scoring low in these quadrants would be consistent with a Dandelion designation. Although preserved interoceptive processing (high IA) could support emotional understanding, abnormally low sensitivity to exteroceptive cues (low Sen, SA) might limit the extent to which external events trigger strong emotions in Lexithymic Dandelions, and clarify why they seek pleasurable stimulation (Seek). Together, these characteristics may explain why members of this group had the lowest levels of depression, anxiety, and stress (Table 5).

Lexithymic Dandelions may have what Moormann et al. (2008) refer to as type III alexithymia—a subtype characterized by intact ability to identify and describe emotions but dampened emotional responses. However, we question whether they have a limited fantasy life. Indeed, we found that Lexithymic Dandelions had average AS scores (Table 5), suggesting that they report typical imagery and dreaming—as might be expected if their average EOT scores indicate a largely preserved ability to direct attention inward and actively maintain vivid images in working memory. We propose that the assumption that type III alexithymia is associated with weak fantasizing may be based on the fact that subtyping studies using the BVAQ have used a composite score (intended to measure “affective” alexithymic traits) that collapses across emotionalizing and fantasizing abilities. Future work specifically investigating mental imagery and fantasy in this group is warranted. Fantasizing abilities might support the use of certain emotion regulation strategies such as distraction (attentional deployment) or imagining that a distressing event happened to someone else or at a different time (an appraisal strategy called *distancing*).

#### Lexithymic Orchids

The Lexithymic Orchids reported below-average DIF/DDF and EOT. Their EOT scores were significantly lower than those of the Modal and Alexithymic Tulip classes (*p* ≤ 0.001), and their mean IA scores were slightly above the mean (*Z* = 0.45). Interestingly, their scores on Sen and SA were higher than those of Lexithymic Dandelions (*p* ≤ 0.001) and higher than their own scores on LR or Seek (*p* ≤ 0.003). Indeed, in all but one case, their scores in Sen and/or SA were unusually high (peaks). Their heightened sensitivity, combined with their tendency to *attend* to their feelings (low EOT), may explain why over half of this group reported moderate levels of anxiety (Table 5). Despite this, most reported few signs of depression or stress, possibly because they have learned ways to understand and manage their emotions.

Overall, the Lexithymic Orchids appear to have a profile most similar to what Moormann et al. (2008) refer to as “lexithymic.” These authors suggest that individuals with this subtype can have a histrionic/dramatic personality style, that they know how to manipulate and make others “like them” (p. 34), and that (consistent with an Orchid designation; Smolewska et al., 2006) they are open to new experiences. Interestingly, Moormann et al. (2008) also report that the perceived emotional warmth of one’s mother predicts membership in this subtype. This may be meaningful as Aron et al. (2012) argue that Orchids who are exposed to supportive early environments are able to develop better emotion regulation skills than those exposed to early adversity. It may be that differences in early life experiences explain why DIF, DDF, and DASS-21 scores of this group were lower than those of Orchids with co-occurring alexithymia (see Figure 3 and Table 5). The combination of heightened sensitivity, moderate anxiety, and good emotion appraisal/regulation seen in Lexithymic Orchids may be quite adaptive, as it could help them spot and react to potential threats effectively.

#### Alexithymic Tulips

Alexithymic Tulips had elevated TAS-20 subscale scores; indeed, a third of them had DIF scores in the high-average range or above (*Z* > 0.66), and approximately half had DDF and EOT scores in this range, suggesting the presence of marked problems with emotional appraisal and strong EOT. This group scored high on Sen and SA, in the average range on LR, and well below average on Seek. Whereas 69% had sensory processing styles characterized by valleys in Seek, this feature was *never* seen in Alexithymic Orchids (described later). Indeed, only the Alexithymic Tulips reported an unusually weak tendency to seek out pleasurable stimulation. This might partially explain why almost two-thirds of them reported mild-to-moderate depression (Table 5).

Almost all of the Alexithymic Tulips (92%) had peaks in the Sen and/or SA quadrants. However, we hypothesize that this group differs from the Alexithymic Orchids (who were also unusually sensitive) in that they weigh exteroceptive cues more strongly than interoceptive or body-based cues. This would explain why their IA was significantly lower than that of Alexithymic Orchids (*p* < 0.001) and why they were, in fact, *the only group to have below average IA* (*Z* = −0.77). This is important to note, given that interoceptive deficits are becoming widely considered a hallmark of alexithymia (Herbert et al., 2011; Brewer et al., 2016; Sowden et al., 2016; Murphy et al., 2017, 2018). It is possible that studies in which alexithymia was related to a tendency to prioritize exteroceptive information when completing an IA task (Murphy et al., 2018) had a preponderance of Alexithymic Tulips and that Alexithymic Orchids were over-represented in studies finding no link between alexithymia and interoceptive deficits (e.g., Nicholson et al., 2018).

We propose that, in Alexithymic Tulips, weak IA reflects the fact that affective states are poorly differentiated from one another. In the model of Bird and Viding (2014), this should make it difficult not only to represent one’s own affective state but also to empathize with another. Indeed, past research suggests that low IA leads to greater instability in the sense of self and a blurring of the lines between self and other (see Lombardo et al., 2010; Tsakiris, 2017) and that both low IA (Shah et al., 2017; Mul et al., 2018) and high EOT (Grynberg et al., 2010; Lyvers et al., 2017) are negatively associated with emotion contagion, affective theory of mind, and empathy. Our prediction that problems with empathy would be common in the Alexithymic Tulips is consistent with the finding of Kajanoja et al. (2017) that individuals displaying an alexithymia subtype characterized by elevated DDF and EOT show empathic deficits. Interestingly, Lane et al. (2015b) argue that the problems with mentally representing emotions in people with an “affective agnosic” form of alexithymia arise from impairments in integrating body-based cues and appraising situations.

If our Alexithymic Tulips are indeed prone to empathic deficits, they would appear to display many features associated with what Moormann et al. (2008) refer to as “type I” alexithymia [or agnosic alexithymia of Lane et al. (2015a)]. However, although Moormann et al. (2008) state that type I alexithymia is *not* associated with anxiety, we found that many of our Alexithymic Tulips reported moderate-to-severe anxiety (Table 5). We suggest that although their hypersensitivity to exteroceptive cues *does* make them susceptible to anxiety, Alexithymic Tulips often cope with this by avoiding situations that make them uncomfortable, avoiding focusing on their emotions and actively suppressing their emotional responses. Engaging in these self-regulatory strategies could make them appear emotionally flat. The idea that they do frequently engage in avoidance is suggested by the fact that 82% of individuals in this class had extremely high SA scores. Overuse of avoidant coping, perhaps especially in social situations, may explain why those with type I alexithymia are often described as being very shy and socially withdrawn (Moormann et al., 2008). It would be interesting in future studies to measure levels of avoidant personality disorder and social anxiety in members of this group.

We conclude this section by highlighting an interesting point, namely that Alexithymic Tulips and Lexithymic Dandelions were the only groups whose scores on *all* of the LPA input variables were significantly different from one another (*p* < 0.001). As noted earlier, Alexithymic Tulips tended to have problems with emotional appraisal (high DIF/DDF) and EOT, reported below-average IA, and scored low on Seek, in the average range on LR, and high on Sen and SA. In contrast, Lexithymic Dandelions reported very few problems with emotional appraisal (low DIF/DDF), had average EOT and above-average IA, and scored high on Seek and low on LR, Sen, and SA. We suggested that the former group may have type I alexithymia and the latter type III alexithymia. Moormann et al. (2008) suggest that individuals with both of these subtypes score low on “emotionalizing” (as assessed by the BVAQ), but we argued above that the *reasons* that they often fail to “show” their emotions might be quite different. Specifically, Alexithymic Tulips may generate atypical representations of their bodily state and actively avoid situations that make them uncomfortable (including thinking about their feelings). In contrast, Lexithymic Dandelions may form and accurately appraise body-based cues but be hyposensitive to external events that trigger strong feelings. These differences, in combination with possible between-class differences in emotional understanding and empathy, could place individuals in these groups at risk for developing distinctly different kinds of personality disorders. Indeed, Moormann et al. (2008) argue that those with type I alexithymia (Alexithymic Tulips) may be at heightened risk for schizoid personality disorder, but that those with type III alexithymia (Lexithymic Dandelions) might be self-focused, emotionally manipulative, and/or antisocial—characteristics that might increase risk for antisocial personality disorder, clinically significant narcissism, or the grandiose form of subclinical narcissism (which is associated with low levels of anxiety and depression; e.g., Sedikides et al., 2004).

#### Alexithymic Orchids

Unlike the Alexithymic Tulips, the Alexithymic Orchids appeared to be principally impaired in their emotion appraisal skills. Thus, their EOT scores were significantly lower than their DIF and DDF scores (*p* ≤ 0.001) and lower than the EOT scores of Alexithymic Tulips (*p* < 0.001). As noted earlier, Alexithymic Orchids had average IA. Their scores on the AASP were uniformly high; thus, whereas their SA and Sen scores were comparable to corresponding scores of Alexithymic Tulips, their LR and Seek scores were higher (*p* ≤ 0.01). Moreover, 70% of individuals in this group had peaks in three or four of the AASP quadrants. In marked contrast, only 36% of Alexithymic Tulips and 14% of Lexithymic Orchids had profiles with more than two peaks.

Why would this group exhibit such extreme AASP profiles? This may relate to how they regulate their responses to stimuli in context. Our data suggest that Alexithymic Orchids—highly sensitive individuals who *attend* to their feelings (average EOT) but have difficulty *making sense* of them (high DIF/DDF)—experience considerable anxiety and stress. Indeed, this group reported the highest rates of severe/extremely severe anxiety and stress, and they obtained the highest average anxiety and stress scores of any group (see Table 5). High anxiety and stress might lead some individuals to actively seek out pleasurable stimulation or to react to negative events with agitation, irritability, and impatience (feelings that could trigger “fight” responses). However, feeling anxious or stressed may also prompt active avoidance or behavioral inhibition that could result in slow responding, “pausing to check” when one encounters a novel situation (a characteristic associated with Orchids; Aron et al., 2012), and “freezing” (Roelofs, 2017). Individuals who experience extreme sensitivity along with competing drives to respond with approach, avoidance, or behavioral inhibition might be expected to score high across all four AASP quadrants. They might also be expected to show strong activation of the behavioral approach and behavioral inhibition systems (see Carver and White, 1994; Gray and McNaugthon, 2000). This latter point is of interest, as SPS has previously been linked to the activation of these systems (Aron and Aron, 1997; Smolewska et al., 2006). Our results suggest that these links might be strongest in individuals with SPS who have co-occurring alexithymia.

Overall, Alexithymic Orchids appear to have a profile most similar to type II alexithymia as described by Moormann et al. (2008). These authors suggest that this subtype may be linked to borderline personality disorder—a personality tendency that is associated with negative mood (anxiety, anger, and sadness), personal relationships marked by conflict and repeated breakups and reconciliations, and insecure attachment (Millon and Davis, 2000; Meyer et al., 2005). Interestingly, type II alexithymia is often seen in women with a history of childhood sexual abuse (Albach et al., 1996; Moormann et al., 1997, 2004). Although we did not gather data on our participants’ early histories, research linking both alexithymia (see Karukivi and Saarijärvi, 2014) and emotional dysregulation in SPS (e.g., Aron et al., 2005) with early adversity suggest that it may have been informative to do so. Factors such as insecure attachment and early abuse may have contributed to difficulties that Alexithymic Orchids had in learning to understand and regulate their emotions. Interestingly, Aron et al., 2012, p. 271) state that Orchids raised in suboptimal environments show “uncontrolled emotional reactivity…[that]…leads to overarousal when conscious decision making is required and inaccurate decisions when faster responses are needed.” This description fits well with the characterization of the Alexithymic Orchids we offered earlier.

As a final note, Moormann et al. (2008) argue that individuals with type I and II alexithymia differ in their tendency to fantasize, with type II individuals having richer fantasy lives. If this is correct, one might predict that Alexithymic Orchids (who we suggest may have a type II profile) would report more vivid mental images and dreams than Alexithymic Tulips. Consistent with this, we found that Alexithymic Orchids had the highest mean AS scores of any class (Table 5), with two-thirds scoring in the high-average range or above. The richness of their inner lives may be attributable, in part, to their ability to turn attention inward and maintain vivid images in working memory. Failure to account for the possibility that there may be two alexithymia subtypes that can be distinguished, in part, on the basis of EOT and fantasy may explain why impaired fantasizing has not been found to be a consistent feature of alexithymia (e.g., Preece et al., 2017).

## Limitations and Future Directions

We opted to investigate IA using self-report because subjective IA in alexithymia has received relatively little research attention and because traditional objective measures of interoceptive abilities such as heartbeat tracking tasks seem to have inherent problems with their reliability and validity. However, alexithymia has also been linked to atypicalities in other areas of interoceptive competence (Longarzo et al., 2015; Scarpazza et al., 2015; Zamariola et al., 2018). It would be interesting to determine if the alexithymia subtypes we identified differ in these domains.

Supplementing the TAS-20 with an interview-based measure or adding objective measures of physiological responsivity, sensory processing, or simulation (e.g., facial mimicry) would be useful in future studies, as would determining how individuals belonging to different subtypes perform on emotion perception tasks—particularly the perception of positive emotions, which have generally been understudied (Shiota et al., 2017). In this regard, we have recently shown that aspects of both alexithymia (DDF and EOT) and SPS (EOE) predict individual differences in how young adults evaluate emotionally valenced scenes (Rigby et al., 2020).

As a final point, although the results of our LPA are quite consistent with the results of some other subtyping studies, the sample size in the present study was relatively small for LPA. We also recognize that our university sample may not be representative of the general population. Given these limitations, replication is needed.

## Conclusion

The results of the present research provide several novel insights and lay the groundwork for future research. The results described in Part A support the view that alexithymia is a multifaceted trait. They also highlight the importance of attending to how strongly individuals with alexithymia endorse EOT, as this varies widely. We extended previous findings by providing a more nuanced view of how particular ways of processing, experiencing, and responding to different situations relate to specific alexithymic traits. We also provided several important insights into the relationships between alexithymic traits, SPS, and IA. Some of this work led us to suggest that low IA may arise from atypical sensory processing/integration that impacts embodiment. Indeed, in Part A, we presented evidence that low IA is just one facet of atypical sensory processing that can characterize certain individuals with alexithymia and that one’s general sensory processing style mediates the relationships between IA and specific alexithymic traits.

An important conclusion from the findings described in Part B was that the subtypes we identified were generally consistent with those described in past research utilizing the BVAQ (Moormann et al., 2008). However, our findings have provided novel information about the sensory processing styles of individuals within these subgroups and about the likelihood that they have co-occurring SPS. Given the fact that the classes were highly distinguishable based on unique combinations of their alexithymia and SPS profiles, researchers who are interested in either of these constructs should consider measuring both; incorporating a measure of early adversity would also be advisable. The fact that low IA was only evident in one of the two subtypes of alexithymia identified in the current study suggests that not *all* individuals with alexithymia experience a “general failure in interoception” (Brewer et al., 2016). We argue that the more universal problems in alexithymia (seen in both Alexithymic Tulips and Alexithymic Orchids) relate to atypicalities in appraisal and emotion regulation.

Although applying a label to a given individual may be useful, as it conveys information about how that individual is *likely* to process and respond to different kinds of stimuli in context, it is important to recognize that traits such as alexithymia and SPS are continuously distributed in the population (Lionetti et al., 2018; Bagby et al., 2020). As noted earlier, one can also see any combination of AASP scores, suggesting that it is important to carefully examine the *pattern* in these scores rather than focusing on any individual quadrant score. For these reasons, we urge researchers and clinicians to regard the subtypes identified here as “prototypes” and to carefully examine individual profiles when interpreting the results of their research studies and clinical assessments. Realizing that the lines between these subtypes are “gray” obviates the need to group together, under the heading of “mixed” types, individuals who have widely discrepant profiles but who do not fit neatly into one of the five subtypes listed earlier (as in Moormann et al., 2008). It is also likely to lead to a better understanding of brain–behavior relationships and to the development of personalized interventions that better support those who are experiencing difficulties in daily life.

## Data Availability Statement

The dataset presented in this study can be found in an online repository. The name of the respository and accession number can be found below: https://doi.org/10.34990/FK2/603TSA.

## Ethics Statement

These studies involved human participants and were reviewed and approved by Psychology/Sociology Research Ethics Board, University of Manitoba. The participants provided their written informed consent to participate in this study.

## Author Contributions

Authors are listed in order based on the importance of their contributions. SR performed data collection and preliminary analyses. SR and LJ were involved in all remaining aspects of the research. Both authors contributed to the article and approved the submitted version.

## Conflict of Interest

The authors declare that the research was conducted in the absence of any commercial or financial relationships that could be construed as a potential conflict of interest.
